# High quality draft genomic sequence of *Flavihumibacter solisilvae* 3-3^T^

**DOI:** 10.1186/s40793-015-0037-6

**Published:** 2015-09-19

**Authors:** Gang Zhou, Chong Chen, Che Ok Jeon, Gejiao Wang, Mingshun Li

**Affiliations:** State Key Laboratory of Agricultural Microbiology, College of Life Sciences and Technology, Huazhong Agricultural University, Wuhan, 430070 People’s Republic of China; Department of Life Science, Chung-Ang University, Seoul, 156-756 Republic of Korea

**Keywords:** *Flavihumibacter*, *Flavihumibacter solisilvae*, Genomic information, Genotype, Phenotype

## Abstract

*Flavihumibacter solisilvae* 3-3^T^ (= KACC 17917^T^ = JCM 19891^T^) represents a type strain of the genus *Flavihumibacter* within the family *Chitinophagaceae*. This strain can use various sole carbon sources, making it applicable in industry and bioremediation. In this study, the draft genomic information of *F. solisilvae* 3-3^T^ is described. *F. solisilvae* 3-3^T^ owns a genome size of 5.41 Mbp, 47 % GC content and a total of 4,698 genes, including 4,215 protein coding genes, 439 pseudo genes and 44 RNA encoding genes. Analysis of its genome reveals high correlation between the genotypes and the phenotypes.

## Introduction

The genus *Flavihumibacter* was established in 2010 [1] and comprises three recognized species, *Flavihumibacter petaseus* T41^T^ [1], *Flavihumibacter cheonanensis* WS16^T^ [2] and *Flavihumibacter solisilvae* 3-3^T^ [3], that were isolated from a subtropical rainforest soil, a shallow stream sediment and a forest soil, respectively. The *Flavihumibacter* members are Gram-positive, rod-shaped, strictly aerobic, non-motile, yellow-pigmented bacteria. The strains all contain phosphatidylethanolamine (as the major polar lipid, menaquinone-7 as the major respiratory quinine, iso-C_15:0_ and iso-C_15:1_ G as the principal fatty acids. In addition, the strains are oxidase- and catalase-positive and with a G + C content range of 45.9-49.5 mol% [1–3].

To the best of our knowledge, the genomic information of *Flavihumibacter* members still remains unknown. In this study, we present the draft genome information of *F. solisilvae* 3-3^T^. A polyphasic taxonomic study revealed that *F. solisilvae* 3-3^T^ could utilize 33 kinds of sole carbon substrates, including 11 kinds of saccharides and 22 kinds of organic acids and amino acids [3]. Specially, this strain could utilize aromatic compound 4-hydroxyphenylacetic acid as a sole carbon source making it applicable environmental bioremediation [4–6]. In addition, this strain could utilize quinic acid as a sole carbon. Quinic acid is the substrate used to synthesize aromatic amino acids (phenylalanine, tyrosine and tryptophan) via the shikimate pathway. These aromatic amino acids are very useful as food additives, sweetener and pharmaceutical intermediates [6, 7]. The genome analysis of *F. solisilvae* 3-3^T^ will provide the genomic basis for better understanding these mechanisms and applying the strain to industries and bioremediation more efficiently.

## Organism information

### Classification and features

**Fig. 1:**
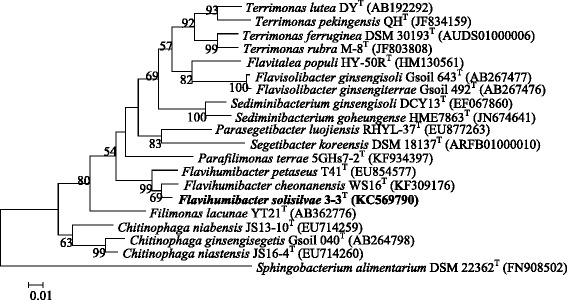
A 16S rRNA gene based ML phylogenetic tree showing the phylogenetic position of *F. solisilvae* 3-3^T^. Bootstrap values (>50 %) based on 1,000 replications are shown at branch nodes. Bar, 1 substitutions per 100 nucleotide positions. *Sphingobacterium alimentarium* DSM 22362^T^ is used as the outgroup. The GenBank accession numbers are shown in parentheses

*F. solisilvae* 3-3^T^ was isolated from forest soil of Bac Kan province in Vietnam [3]. The classification and features of *F. solisilvae* 3-3^T^ are shown in Table 1. A maximum-likelihood tree was constructed based on the 16S rRNA gene sequences using MEGA 5.0 [8]. The bootstrap values were calculated based on 1,000 replications and distances were calculated in accordance with Kimura’s two-parameter method [9]. The phylogenetic tree showed that *F. solisilvae* 3-3^T^ was clustered with the other *Flavihumibacter* members (Fig. 1).

**Table 1:** Classification and general features of *F. solisilvae* 3-3^T^ according to the MIGS recommendations [22]

MIGS ID	Property	Term	Evidence code^a^
	Classification	Domain *Bacteria*	TAS [23]
		Phylum *Bacteroidetes*	TAS [24, 25]
		Class *Sphingobacteriia*	TAS [25, 26]
		Order *Sphingobacteriales*	TAS [25, 27]
		Family *Chitinophagaceae*	TAS [28]
		Genus *Flavihumibacter*	TAS [1]
		Species *Flavihumibacter solisilvae*	TAS [3]
		Strain: 3-3^T^	TAS [3]
	Gram stain	Positive	TAS [3]
	Cell shape	Rod-shaped	TAS [3]
	Motility	Non-motile	TAS [3]
	Sporulation	Non-sporulating	NAS
	Temperature range	20–37 °C	TAS [3]
	Optimum temperature	28 °C	TAS [3]
	pH range; Optimum	5.5–9.5; 7.5	TAS [3]
	Carbon source	Sucrose, D-glucose, D-galactose, lactose, N-acetyl-glucosamine, L-arabinose, D-maltose, glycerol, dextrin, D-melibiose, glucuronamide, succinic acid mono-methyl ester, L-aspartic acid, D-galacturonic acid, D-glucosaminic acid, D-glucuronic acid, malonic acid, β-hydroxybutyric acid, 4-hydroxyphenlyacetic acid, quinic acid, D-saccharic acid, bromosuccinic acid, succinamic acid, L-pyroglutamic acid, urocanic acid,γ-aminobutyric acid, L-alanine, L-serine, D-alanine, L-histidine, L-alanyl-glycine, L-alaninamide and L-asparagine.	TAS [3]
GS-6	Habitat	Forest soil	TAS [3]
MIGS-6.3	Salinity	0–0.5 % NaCl (w/v)	TAS [3]
MIGS-22	Oxygen requirement	Aerobic	TAS [3]
MIGS-15	Biotic relationship	Free-living	NAS
MIGS-14	Pathogenicity	Non-pathogenic	NAS
MIGS-4	Geographic location	Bac Kan Province, Viet Nam	TAS [3]
MIGS-5	Sample collection	2012	TAS [3]
MIGS-4.1	Latitude	Not reported	
MIGS-4.2	Longitude	Not reported	
MIGS-4.4	Altitude	Not reported	

^a^Evidence codes - IDA: Inferred from Direct Assay; TAS: Traceable Author Statement (i.e., a direct report exists in the literature); NAS: Non-traceable Author Statement (i.e., not directly observed for the living, isolated sample, but based on a generally accepted property for the species, or anecdotal evidence). These evidence codes are from the Gene Ontology project [29]

**Fig. 2:**
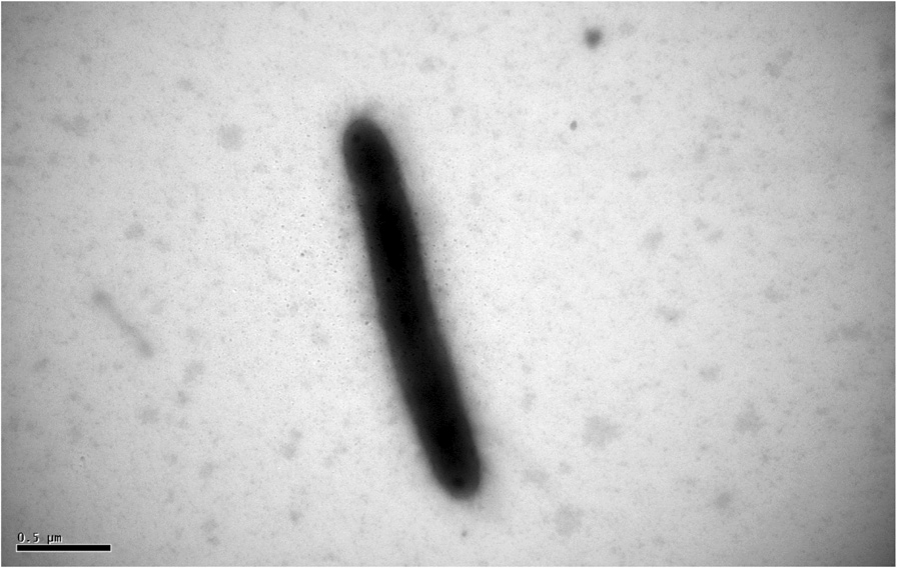
A transmission electron micrograph of *F. solisilvae* 3-3^T^ cell. The bar indicates 0.5 μm

Cells of *F. solisilvae* 3-3^T^ (Fig. 2) are Gram-positive, aerobic, non-motile, and rod-shaped. Colony is yellow due to the production of flexirubin-type pigments [10]. *F. solisilvae* 3-3^T^ grows well on NA and R2A agar (optimum), but do not grow on LB or TSA agar [3]. It can hydrolyze aesulin, gelatin, casein and tyrosine [3]. *F. solisilvae* 3-3^T^ can also utilize various carbohydrate substrates (Table 1) and produces several glycosyl hydrolases, such as β-N-acetylhexosaminidase, α-galactosidase, β-glucosidase, β-galactosidase, α-fucosidase, α-mannosidase and α-glucosidase [3].

*F. solisilvae* 3-3^T^ contains iso-C_15:0_, iso-C_15:1_ G and summed feature 3 (C_16:1_*ω*6*c*/C_16:1_*ω*7*c*) as the principal fatty acids, MK-7 as the major respiratory quinine. The major polar lipids were PE, three unidentified aminolipids and three unidentified lipids [3].

## Genome sequencing information

### Genome project history

*F. solisilvae* 3-3^T^ was selected for sequencing based on its taxonomic representativeness and the potential application in food industry and bioremediation. The genome of *F. solisilvae* 3-3^T^ was sequenced at Wuhan Bio-Broad Co., Ltd, Hubei, China. This Whole Genome Shotgun project has been deposited at DDBJ/EMBL/GenBank under the accession JSVC00000000. The version described in this paper is version JSVC00000000.1. A summary of the genomic sequencing project information is given in Table 2.

**Table 2:** Project information of *F. solisilvae* 3-3^T^

MIGS ID	Property	Term
MIGS-31	Finishing quality	High-quality draft
MIGS-28	Libraries used	Illumina Paired-End library(300 bp insert size)
MIGS-29	Sequencing platforms	Illumina Hiseq 2000
MIGS-31.2	Fold coverage	250 ×
MIGS-30	Assemblers	velvet v.1.2.10
MIGS-32	Gene calling method	GeneMarkS^+^
	Locus Tag	OI18
	Genbank ID	JSVC00000000
	Genbank Date of Release	2015-01-05
	BIOPROJECT	PRJNA265817
MIGS-13	Source Material Identifier	*F. solisilvae* 3-3^T^ (= KACC 17917^T^ = JCM 19891^T^)
	Project relevance	Genome comparison

### Growth conditions and genomic DNA preparation

*F. solisilvae* 3-3^T^ was grown aerobically in 50 ml R2A broth at 28 °C for 24 h with 160 r/min shaking. About 20 mg cells were harvested by centrifugation and suspended in normal saline, and then lysed using lysozyme. The DNA was obtained using the QiAamp kit according to the manufacturer’s instruction (Qiagen, Germany).

### Genome sequencing and assembly

The genome of *F. solisilvae* 3-3^T^ was sequenced by Illumina Hiseq 2,000 technology [11] with Paired-End library strategy (300 bp insert size). TruSeq DNA Sample Preparation Kits are used to prepare DNA libraries with insert sizes from 300–500 bp for single, paired-end, and multiplexed sequencing. The protocol supports shearing by either sonication or nebulization of 1 μg of DNA [12]. The genome of *F. solisilvae* 3-3^T^ generated 7,041,525 reads totaling 1,422,388,050 bp data with an average coverage of 250 ×. Sequence assembly and quality assessment were performed using velvet v.1.2.10 [13] software. Finally, all reads were assembled into 75 contigs (> 200 bp) with a genome size of 5.41 Mbp.

### Genome annotation

Genome annotation was performed through the NCBI Prokaryotic Genome Annotation Pipeline which combined the Best-Placed reference protein set and the gene caller GeneMarkS+. WebMGA-server [14] with E-value cutoff 1-e^3^ was used to assess the COGs. The translated predicted CDSs were also used to search against the Pfam protein families database [15]. TMHMM Server v.2.0 [16], SignalP 4.1 Server [17] and CRISPRfinder program [18] were used to predict transmenbrane helices, signal peptides and CRISPRs in the genome, respectively. The metabolic pathway analysis were constructed using the KEGG (Kyoto Encyclopedia of Genes and Genomes) [19].

## Genome properties

**Fig. 3:**
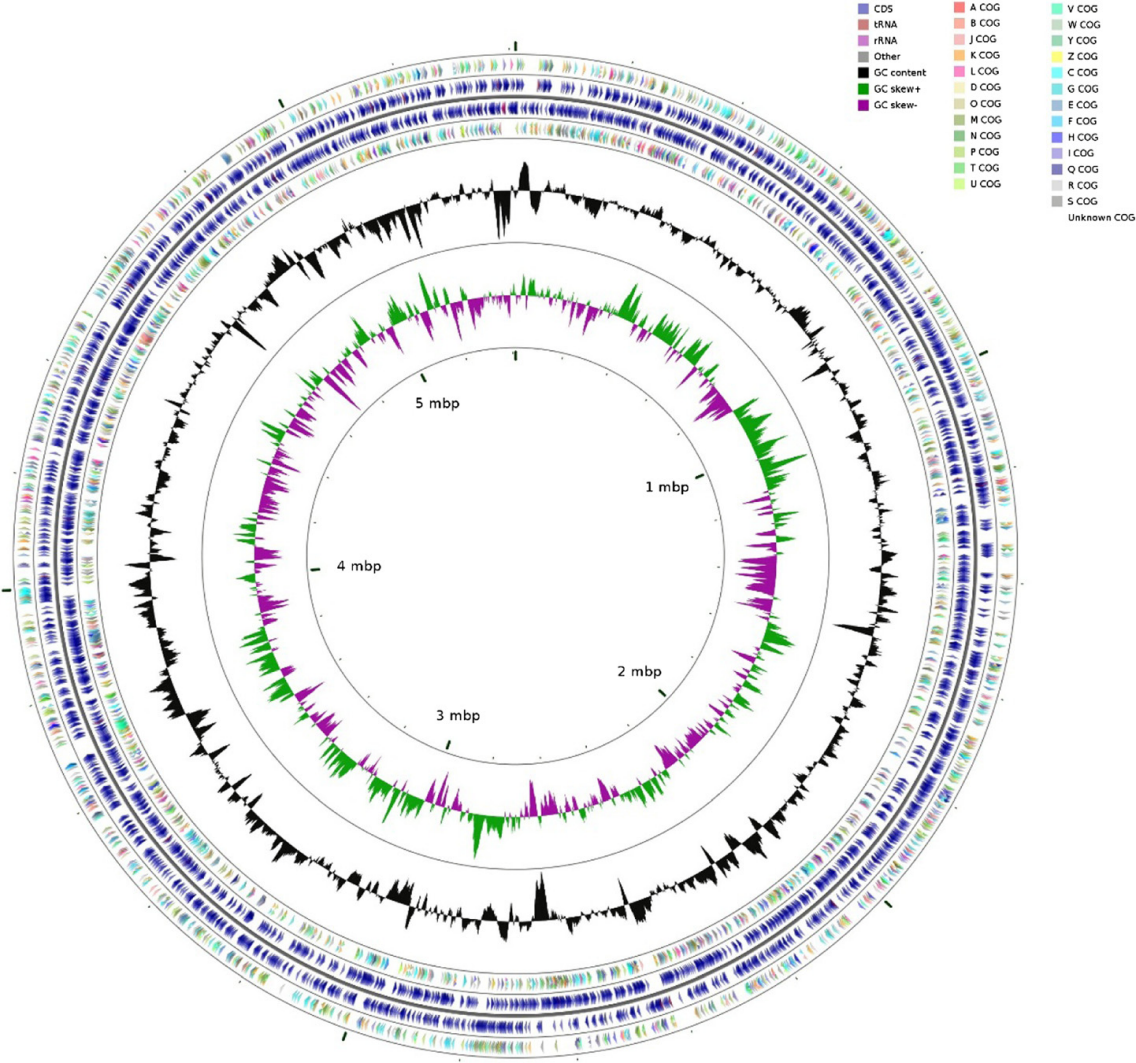
A Graphical circular map of *F. solisilvae* 3-3^T^ genome. From outside to center, ring 1, 4 show protein-coding genes colored by COG categories on forward or reverse strand; ring 2, 3 denote genes on forward or reverse strand; ring 5 shows G + C content plot, and the innermost ring represents GC skew

The daft genome size of *F. solisilvae* 3-3^T^ is 5,410,659 bp with 47 % GC content and contains 75 contigs. From a total of 4,698 genes, 4,215 (89.72 %) genes are protein coding genes, 439 (9.34 %) are pseudo genes and 44 (0.94 %) are RNA encoding genes. The genome properties and statistics are shown in Table 3 and Fig. 3. Altogether, 3,137 (74.42 %) protein coding genes are distributed into COG functional categories (Table 4).

**Table 3:** Genome statistics of *F. solisilvae* 3-3^T^

Attribute	Value	% of total^a^
Genome size (bp)	5,410,659	100.00
DNA coding (bp)	4,540,989	83.93
DNA G + C (bp)	2,543,035	47.00
DNA scaffolds	75	-
Total genes	4,698	100.00
Protein coding genes	4,215	89.72
RNA genes	44	0.94
Pseudo genes	439	9.34
Frameshifted Genes	9	0.19
Genes with function prediction	1,893	44.91
Genes assigned to COGs	3,137	74.42
Genes with Pfam domains	3,511	83.30
Genes with signal peptides	670	15.89
Genes with transmembrane helices	919	21.80
CRISPR repeats	1	-

^a^The total is based on either the size of the genome in base pairs or the total number of protein coding genes in the annotated genome

**Table 4:** Number of genes associated with general COG functional categories of *F. solisilvae* 3-3^T^ genome

Code	Value	% age	Description
J	169	4.01	Translation, ribosomal structure and biogenesis
A	0	0.00	RNA processing and modification
K	278	6.60	Transcription
L	115	2.73	Replication, recombination and repair
B	2	0.05	Chromatin structure and dynamics
D	23	0.55	Cell cycle control, cell division, chromosome partitioning
V	93	2.21	Defense mechanisms
T	243	5.77	Signal transduction mechanisms
M	251	5.95	Cell wall/membrane/envelope biogenesis
N	9	0.21	Cell motility
U	58	1.38	Intracellular trafficking and secretion
O	110	2.61	Posttranslational modification, protein turnover, chaperones
C	206	4.89	Energy production and conversion
G	224	5.31	Carbohydrate transport and metabolism
E	239	5.67	Amino acid transport and metabolism
F	76	1.80	Nucleotide transport and metabolism
H	155	3.68	Coenzyme transport and metabolism
I	129	3.06	Lipid transport and metabolism
P	205	4.86	Inorganic ion transport and metabolism
Q	87	2.06	Secondary metabolites biosynthesis, transport and catabolism
R	466	11.06	General function prediction only
S	315	7.47	Function unknown
-	1078	25.58	Not in COGS

The total is based on the total number of protein coding genes in the genome

## Insights from the genome sequence

*F. solisilvae* 3-3^T^ could grow on 33 kinds of sole carbon substrates including saccharides, organic acids and amino acids [3] (Table 1). Analysis of the genome reveals that this strain possesses putative enzymes for central carbohydrate metabolism to assimilate these carbon sources through different metabolic pathways [20]. The putative enzymes that responsible to the utilization of 20 sole carbons were found in the genome (Table 5). All key enzymes in the Embden-Meyerhof-Parnas pathway (glucokinase, pyruvate kinase and 6-phosphofructokinase) and TCA cycle are present in *F. solisilvae* 3-3^T^. The key enzymes of Pentose Phosphate pathway (glucose-6-phosphate dehydrogenase, 6-phosphogluconolactonase and 6-phosphogluconate dehydrogenase) were also found.

**Table 5:** Putative enzymes responsible the utilization of different sole carbon sources in the genome of *F. solisilvae* 3-3^T^

Substrates	Enzymes	Accession no.
Sucrose	Alpha-glucosidase	KIC96373
D-maltose	Alpha-glucosidase	KIC96373
D-glucose	Glucokinase	KIC93940
D-galactose	Aldose epimerase	KIC96300
	Galactokinase	KIC95381
Lactose	Beta-galactosidase	KIC94337
Glycerol	Glycerol kinase	KIC93992
	Glycerol-3-phosphate dehydrogenase	KIC94583
N-acetyl-glucosamine	β-N-acetylhexosaminidase	KIC92674
L-arabinose	Arabinose isomerase	KIC96297
D-melibiose	Alpha-galactosidase	KIC96021
4-hydroxyphenlyacetic acid	4-hydroxyphenylpyruvate dioxygenase	KIC95062
	Homogentisate 1,2-dioxygenase	KIC93392
Quinic acid	3-dehydroquinate dehydratase	KIC93382
	Shikimate dehydrogenase	KIC92987
	Shikimate kinase	KIC93265
	3-phosphoshikimate 1-carboxyvinyltransferase	KIC94147
	Chorismate synthase	KIC94148
Urocanic acid	Urocanate hydratase	KIC93805
L-asparagine	L-asparaginase	KIC93060
L-aspartic acid	Aspartate ammonia-lyase	KIC93059
L-histidine	Histidine ammonia-lyase	KIC93735
L-serine	Serine dehydratase	KIC94326
L-alanine	Alanine dehydrogenase	KIC92870
D-alanine	D-alanine--D-alanine ligase	KIC93315
D-glucuronic acid	Glucuronate isomerase	KIC95816
D-galacturonic acid	Glucuronate isomerase	KIC95816

The presence of 4-hydroxyphenylpyruvate dioxygenase (KIC95062), homogentisate 1,2-dioxygenase (KIC93392) and other related enzymes suggests that 4-hydroxyphenylacetic acid is degradable via homogentisic acid pathway [21]. In addition, the presence of 3-dehydroquinate dehydratase (KIC93382), shikimate dehydrogenase (KIC92987), shikimate kinase (KIC93265), 3-phosphoshikimate 1-carboxyvinyltransferase (KIC94147) and chorismate synthase (KIC94148) indicates that *F. solisilvae* 3-3^T^ could probably utilize quinic acid to synthesize the three aromatic amino acids (tryptophan, tyrosine and phenylalanine) via shikimate pathway [7].

## Conclusion

To the best of our knowledge, this report provides the first genomic information of the genus *Flavihumibacter*. Analysis of the genome shows high correlation between the genotypes and the phenotypes. The genome possesses many key proteins of central carbohydrate metabolism which provides the genomic basis to utilize the various carbon sources. In addition, analyzing its genome indicates that this strain has potential application for the production of aromatic amino acids and for environmental bioremediation.

## Abbreviations

TCA: Tricarboxylic acid cycle
PE: Phosphatidylethanolamine
MK-7: Menaquinone-7
PGAP: Prokaryotic genome annotation pipeline
